# P50 Bismuth-based β-lactamase inhibitors to combat antibiotic resistance

**DOI:** 10.1093/jacamr/dlaf230.057

**Published:** 2025-12-04

**Authors:** Adedolapo Ajiboye, Jody Winter, Sophie Benjamin

**Affiliations:** Department of Chemistry and Forensics, Nottingham Trent University, Nottingham, UK; Department of Biosciences, Nottingham Trent University, Nottingham, UK; Department of Chemistry and Forensics, Nottingham Trent University, Nottingham, UK

## Abstract

**Background:**

There is an urgent need for the development of new MBL inhibitors. β-Lactamase enzymes are a key mechanism of bacterial resistance against the widely used β-lactam class of antibiotics. While serine β-lactamase inhibitors such as clavulanic acid and sulbactam are available in the clinic, MBLs currently have no commercially available inhibitors.^1^ Bismuth is a generally non-toxic metal that may hold potential for developing MBL inhibitors. There is evidence that bismuth can displace zinc in the MBL active site, leaving it unable to hydrolyse β-lactam substrates.^2^ However, bismuth compounds often struggle with poor solubility and/or bioavailability.

**Objectives:**

The aim of this project is to synthesize and characterize novel molecular, soluble and tuneable bismuth based MBL inhibitors.

**Methods:**

We have synthesized and characterized a library of Bi(iii) complexes, using dipicolinic acid (DPA) and substituted DPA ligands via simple one-pot syntheses. These compounds have been characterized by various methods including FTIR, 1H NMR and single crystal x-ray diffraction. We have also done preliminary MBL inhibition and cell viability assays.

**Results and discussion:**

The synthesized complexes have diverse solid-state structures, determined using single crystal X-ray diffraction. They are water soluble, with some showing greater MBL inhibition ability when compared to colloidal bismuth subcitrate (CBS), an approved drug. The toxicity of some of these compounds are also comparable to CBS.

**Conclusions:**

Although more testing is required. Current results suggest that some of the synthesized compounds are able to inhibit MBLs and restore susceptibility to some antibiotics.
